# Physiotherapy to Alleviate Chest Complications in Acute Pancreatitis With Comorbidities: A Rare Case of Young Female

**DOI:** 10.7759/cureus.62000

**Published:** 2024-06-09

**Authors:** Chitwan S Agrawal, Vaishnavi Yadav, Dhanshri Nikhade

**Affiliations:** 1 Department of Cardiovascular and Respiratory Physiotherapy, Ravi Nair Physiotherapy College, Datta Meghe Institute of Higher Education and Research, Wardha, IND

**Keywords:** chest physiotherapy, ishchemic heart disease, pleural effusion, systemic inflammatory response syndrome, acute pancreatitis, physiotherapy

## Abstract

An abnormal buildup of pleural fluid, known as a pleural effusion, results from an imbalance between excessive formation and absorption. Despite the wide range of pleural effusion causes, including pneumonia, congestive heart failure, and cancer, the majority of cases are attributed to pleural fluid buildup. Acute pancreatitis also leads to complications such as systemic inflammatory response syndrome. A complex pathophysiologic reaction to a range of wounds, including trauma and infections, burns, and pancreatitis, is known as systemic inflammatory response syndrome. It was recognized that a variety of injuries exhibited a similar inflammatory response, making them prime candidates for new anti-inflammatory molecules designed to stop the spread of inflammation or provide targeted therapy. Localized inflammation, a protective response that the body regulates at the site of the injury, can, if lost or overly activated, result in a heightened systemic response known as systemic inflammatory response syndrome. The patient is a 19-year-old female who arrived at Acharya Vinoba Bhave Rural Hospital with complaints of abdominal pain for eight days, abdominal distension for three to four days, breathing difficulty for three to four days, and fever. According to the patient's condition, she was unable to perform normal activities of daily living for eight days. She had breathlessness for eight days, which worsened four days ago. She was diagnosed with pleural effusion, acute pancreatitis, and systemic inflammatory response syndrome. This case is unique as the patient is very young and she has multiple health issues such as severe pancreatitis, ischemic heart disease, systemic inflammatory response syndrome, pulmonary consolidation, and pleural effusion at the same time which makes this condition critical. This study aimed to identify the improvement in this patient after getting physiotherapy treatment. Physiotherapy treatment included lifestyle modifications to reduce weight, performing exercise on a daily basis, breathing exercises airway clearance technique, volumetric incentive spirometer segmental expansion, inspiratory muscle training, chest mobilization, chest proprioceptive neuromuscular facilitation (PNF), and graded mobilization to improve patient condition. When added to standard care, a physiotherapy program improves radiological results, spirometric parameters, and hospital stays in pleural effusion patients.

## Introduction

Severe pancreatitis is frequently associated with a number of other comorbidities; for example, systemic inflammatory response syndrome. The precise causes are still unknown. The main factor contributing to fatality in this situation is multiple organ dysfunction syndrome, which can result from excessive systemic inflammatory response syndrome and damage to distant organs [1]. Rapid identification, severity stratification, fluid control, and nutrition are all essential for providing effective therapy for acute pancreatitis [2]. Gallstones that damage the distal common bile-pancreatic duct are the main cause of acute pancreatitis. If confirmed, decompression of pancreatic duct pressure is recommended for better results, which may improve conventional therapeutic approaches to acute pancreatitis [3].

The term ischemic heart disease is suggested by the study to characterize the differences in heart disease between both genders. In comparison to males, females have a reduced incidence of obstructive coronary artery disease but higher symptoms, ischemia, and worse outcomes [4]. Younger males are at higher risk of ischemic heart disease with elevated plasma fibrinogen levels, and smoking is likely a mediator of this relationship between smoking and ischemic heart disease. The coagulation system and cholesterol metabolism may be affected by the metabolic imbalance that causes ischemic heart disease [5]. Age, smoking history, white blood cell count, viscosity, and fibrinogen are all relevant variables in a model that forecasts ischemic heart disease [6]. This study shows how a gender-specific strategy can help females with heart disease, who frequently deal with considerable psychosocial load, feel less stressed and exhausted. Quality of life and psychological health are the main concerns, particularly as post-coronary event survival rates increase. The effects on proximal and distal targets will be investigated in later follow-ups [7]. The rehabilitation programs for ischemic heart disease patients included the use of neuromuscular manual techniques to improve the functional status of the cardiorespiratory system [8]. Because of heart cachexia and pancreatic exocrine insufficiency, chronic pancreatitis and congestive heart failure are associated with clinical conditions such as sarcopenia, cachexia, and malnutrition. Nonetheless, persistent pancreatitis might also function as a stand-alone risk factor for the development of cardiovascular disease. Malnutrition and cachexia result from long-term pancreatic tissue insult in heart failure patients, and the likelihood of cardiovascular diseases is elevated by exocrine insufficiency and chronic pancreatitis [9].

According to a study by Singh et al., patients who have acute pancreatitis on the day of treatment are more likely to have systemic inflammatory response syndrome, which is present in the majority of hospitalized patients. On day one, patients who meet three or four requirements for systemic inflammatory response syndrome possess worse acute pancreatitis than patients who do not meet systemic inflammatory response syndrome criteria [10]. According to a study by Hirota et al., most patients with acute pancreatitis acquire systemic inflammatory response syndrome, which is a severe condition. Proinflammatory cytokines, which can be released when the Toll-like receptor is activated, cause this syndrome. Cytokines are produced as part of the body's defense mechanism, but too many of them can damage the immune system or cause organ failure by suppressing the immune system [11].

Clinical manifestations of systemic inflammatory response syndrome include a high body temperature, hypothermia, tachycardia, tachypnea, and an alteration in the blood leucocyte count [12]. According to several studies, failed organs, inflammation, and infection lead to organ malfunction and failure, which increases death rates in severe organ failure (systemic inflammatory response syndrome) and modified organ failure cases, which can range from 30% to 80%. The risk factors for modified organ failure must be addressed early [13]. Despite the seriousness of the illness, the emergence of systemic inflammatory response syndrome, and the progression of organ failure, the intensive care unit is routinely referred to for severely ill patients due to respiratory failure [14]. Early detection of systemic inflammatory response syndrome and knowledge of sepsis risk could drastically change clinical practice and possibly result in the administration of advanced, goal-oriented sepsis therapy [15]. In critical care units, oxidative stress is more severe among those with systemic inflammatory response syndrome than in patients without the illness [16].

Both pulmonary consolidation and pleural effusion are frequent in acute pancreatitis, which correlates with its seriousness, providing early warning of severe acute pancreatitis and organ failure [17]. Dyspnea, a dry cough, and chest pain are common symptoms of excessive fluid accumulation in the space between the lungs [18]. The space between the lungs is filled with more fluid than is expelled, a condition known as pleural effusion. This condition is frequently brought on by conditions like raised interstitial fluid, reduced intrapleural pressure, hypoalbuminemia, thoracic duct rupture, diaphragmatic defects, elevated permeability of the pleural membrane, and limited lymphatic flow. Pulmonary embolism, cancer, pneumonia, tuberculosis, and heart failure are common causes in adults. In contrast, pneumonia is the main cause in children. Pleural effusion can be caused by numerous illnesses [19].

Physiotherapy treatment included lifestyle modifications to reduce weight, performing exercise on a daily basis, breathing exercises airway clearance technique, volumetric incentive spirometer segmental expansion, inspiratory muscle training, chest mobilization, chest PNF, and graded mobilization to improve patient condition.

## Case presentation

Patient information

A 19-year-old female arrived at the hospital on September 22 with complaints of abdominal pain for eight days, abdominal distension for three to four days, breathing difficulty for three to four days, and fever. According to the patient's condition, she was unable to perform normal activities of daily living, such as walking, eating, etc. for eight days. She had breathlessness for eight days, which worsened four days ago. During the investigation, the patient was admitted to the medicine intensive care unit and was diagnosed with acute pancreatitis, pleural effusion, systemic inflammatory response syndrome, and also ischemic heart disease. She was experiencing dyspnea for one month, which was typically severe grade 4 when using Modified Medical Research Council scale at rest. The patient was then admitted to the medicine intensive care unit and put on a mechanical ventilator via a bilevel positive airway pressure mask; the fraction of inspired oxygen was 40%, and the positive end-expiratory pressure was 6 cm of H_2_O.

Clinical findings

The patient was endomorphic as she had a height of 5 feet 1 inch with a weight of 72 kg, BMI was 30, waist hip ratio was 0.95 and alert, with a heart rate of 104 beats per minute, blood pressure of 124/86 mmHg, respiratory rate of 22 breaths per minute, and oxygen saturation of 96%. However, chest movement was reduced due to increased respiration work, suggesting tachypnic massive accessory muscle use. Chest auscultation showed reduced air entry in the lower zones. Table 1 shows the timeline of the events.

**Table 1:** Timeline of patients' date of admission and examination.

Episode	Date of episode
Date of admission	September 22, 2023
Date of examination	September 25, 2023

Physiotherapy intervention

**Figure 1:**
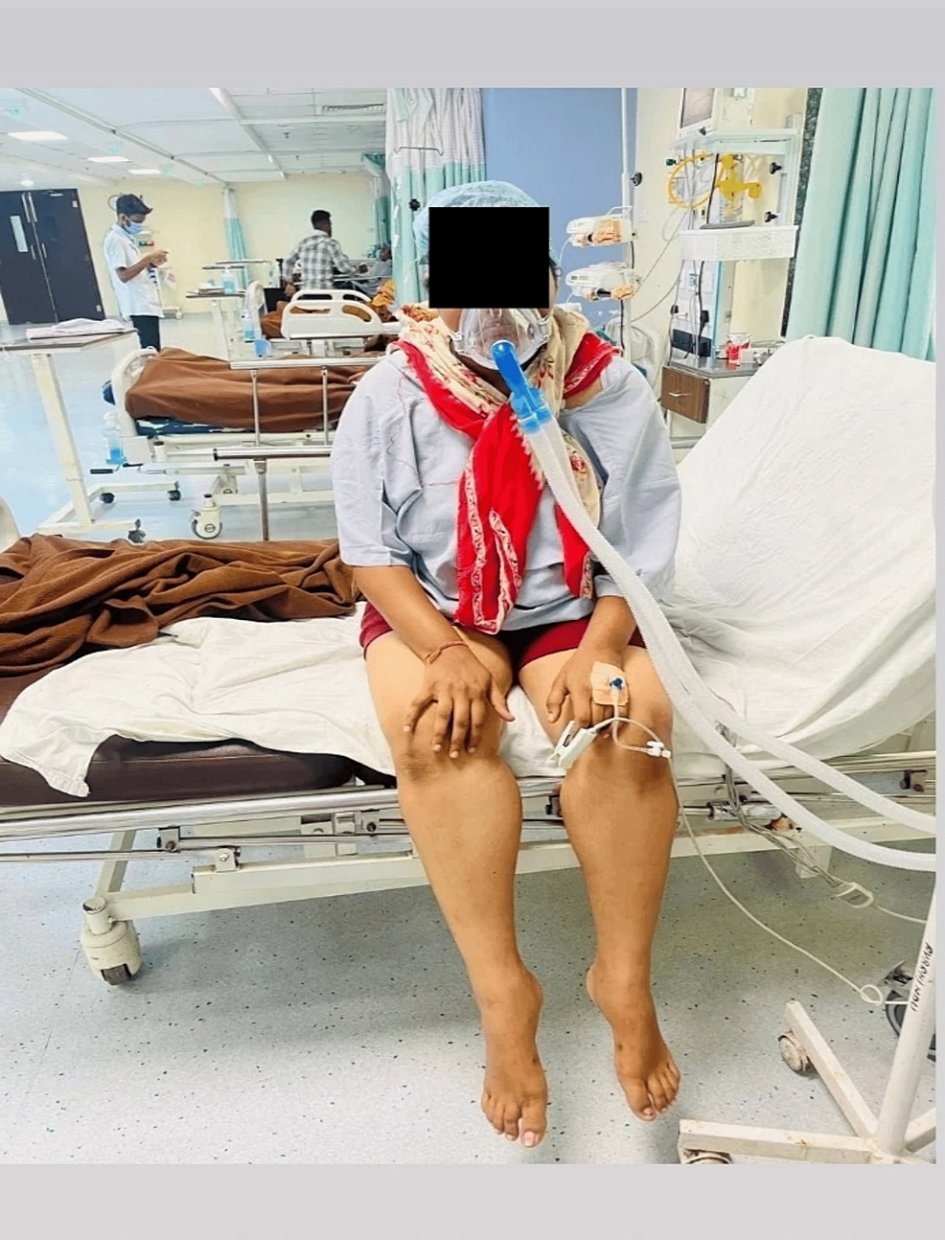
Bedside sitting - patient is sitting on the side of the bed.

**Figure 2:**
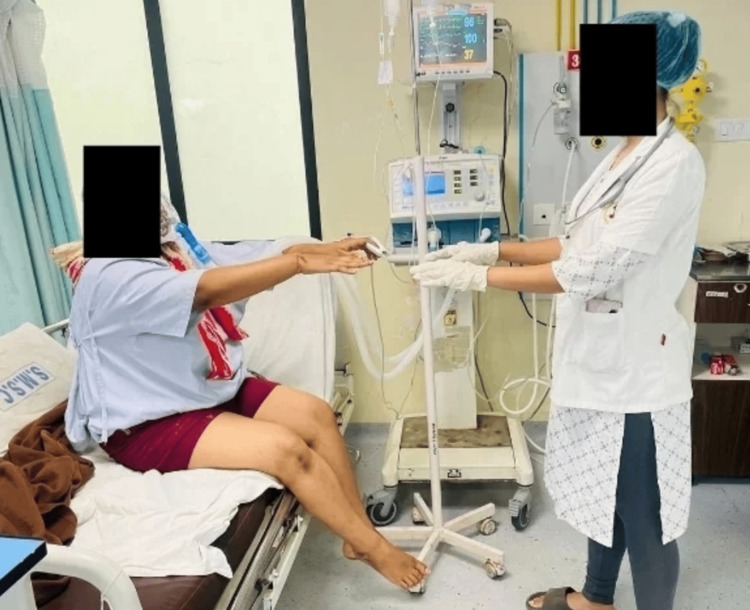
Thoracic expansion - patient performing thoracic expansion.

**Figure 3:**
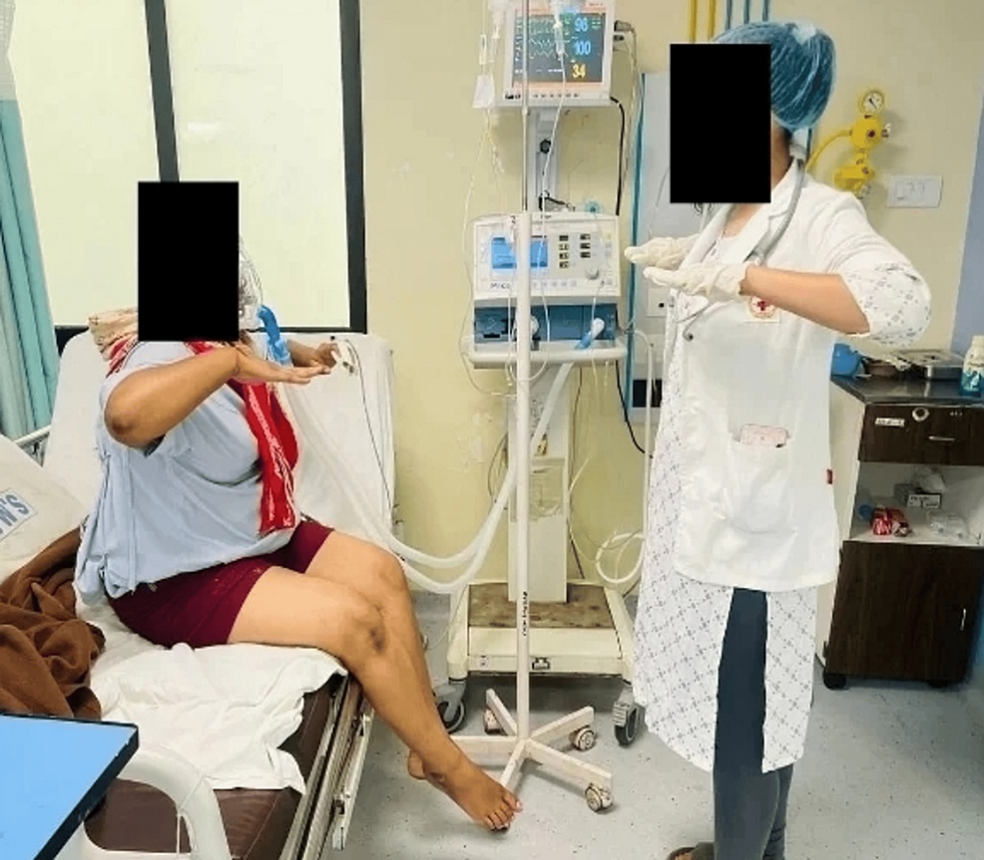
Scapular sets - patients performing scapular bracing.

Table 2 gives information on the physiotherapy intervention received by the patient. The patient's treatment regime is shown in Figures 1-3.

**Table 2:** Physiotherapy management along with its goals and rationale. PNF: proprioceptive neuromuscular facilitation

Goal	Intervention	Rationale
To prevent pulmonary complication	Breathing exercises	To boost oxygen levels and encourage the diaphragm to resume aiding in breathing
To prevent circulatory complication	Ankle pumps	To maintain blood circulation in your legs which can aid in preventing blood clots
To improve lung volume and capacity	Volumetric incentive spirometer segmental expansion	Clearing mucus and secretions from the chest and lungs as well as strengthening and maintaining inflated lungs are all important steps in preventing lung infections
To improve strength of respiratory muscle	Inspiratory muscle training using threshold inspiratory muscle trainer	Improving respiratory muscle function and inspiratory muscle strength may help lower dyspnea during exercise
To improve chest mobility	Chest mobilization, chest PNF, such as perioral stimulation	The respiratory muscles receive proprioceptive feedback from the system, which causes reflexive respiratory movement responses that quicken and deepen breathing
To improve mobility	Graded mobilization	It helps regain a normal breathing pattern enhancing respiratory muscle performance

Outcome measures

Table 3 depicts pre- and post-rehabilitation findings of treatment outcomes.

**Table 3:** Pre- and post-rehabilitation findings of physiotherapy treatment outcomes. ICU: intensive care unit; DASS: Depression Anxiety Stress Scales

Outcome	First day of referral	On the day of discharge	At the time of follow-up
ICU mobility scale score	3	9	10
DASS 21 scale	Moderate	Mild	Mild

## Discussion

Acute pancreatitis is a serious condition that causes high morbidity and mortality. Recent research has clarified the functions of enteral feeding, fine-needle aspiration, prophylactic antibiotics, endoscopic retrograde cholangiopancreatography, and computed tomography in the treatment of the condition [20]. Atherosclerosis of the coronary arteries, which is a dynamic process in ischemic heart disease, can be determined by lifestyle choices, medications, and revascularization, which may result in disease stabilization or regression [21]. The systemic inflammatory response syndrome requirements for fatal sepsis did not work to establish a threshold for death risk [22]. With further refinement, the initial description of systemic inflammatory response syndrome aimed to achieve high sensitivity and specificity by basing it on frequent clinical and laboratory abnormalities [23]. Systemic inflammatory response syndrome is the body's attempt to protect itself from noxious stressors like infection, trauma, surgery, or cancer. When this happens, acute-phase reactants are released, which in turn cause significant changes in the subject's autonomic, endocrine, hematological, and immunological systems.

Based on the fluid's biochemical properties, which typically point to its physiologic formation mechanism, pleural effusions can be divided into transudates and exudates. Transudates are primarily brought on by cirrhosis and heart failure, and imbalances in the hydrostatic and oncotic forces cause them. Nephrotic disorder, atelectasis, peritoneal dialysis, constrictive pericarditis, superior vena cava blockage, and urinothorax are some less frequent causes. Transudative effusions typically disappear after treatment [19]. Exudates, which are primarily brought on by pneumonia, cancer, and thromboembolism, are difficult to diagnose because they change the local factors that affect the buildup of pleural fluid [24]. To determine whether a patient has an exudative or transudative pleural effusion, one should apply Light's criteria. Treatment for underlying diseases like congestive heart failure, cirrhosis, or nephrosis should be the main focus if the condition is transudative. Pleurodesis with a sclerosant may be an option in cases of persistent transudative effusion that results in severe dyspnea. If exudative, the underlying cause should be identified [25].

The goal of the patient's physiotherapy treatment plan was to enhance both general health and respiratory function. It included daily exercise to increase physical fitness and lifestyle adjustments for weight loss. Breathing exercises, airway clearance, segmental expansion, inspiratory muscle training, manual therapies, and graded mobilization strategies were among the respiratory-focused techniques used in the intervention. These methods were applied to improve breathing mechanics and maximize mobility of the chest wall. To help the patient's condition gradually get better, manual therapies and graded mobilization techniques were added to the treatment. Optimizing respiratory function, reducing symptoms, and advancing general health and wellness were the objectives.

## Conclusions

Acute pancreatitis is a condition associated with other co-morbid conditions like heart disease and systemic inflammatory response syndrome and also leads to chest complications like pleural effusion. Making the patient independent and able to carry out daily activities is possible by giving chest physiotherapy, including chest mobilization, volumetric incentive spirometer, segmental expansion, inspiratory muscle training, and changing lifestyle habits. The patient's physiotherapy treatment plan aimed to enhance respiratory function and overall health through daily exercise, dietary changes, breathing exercises, airway clearance, and manual therapies. Breathing improvement methods included segmental expansion and inspiratory muscle training. By promoting holistic well-being, reducing symptoms, and optimizing function, the approach demonstrated patient-centered care.
